# Prediction of Malignant Acute Middle Cerebral Artery Infarction via Computed Tomography Radiomics

**DOI:** 10.3389/fnins.2020.00708

**Published:** 2020-07-07

**Authors:** Xuehua Wen, Yumei Li, Xiaodong He, Yuyun Xu, Zhenyu Shu, Xingfei Hu, Junfa Chen, Hongyang Jiang, Xiangyang Gong

**Affiliations:** ^1^Department of Radiology, Zhejiang Provincial People’s Hospital, Affiliated People’s Hospital of Hangzhou Medical College, Hangzhou, China; ^2^Institute of Artificial Intelligence and Remote Imaging, Hangzhou Medical College, Hangzhou, China

**Keywords:** stroke, malignant, middle cerebral artery, computed tomography, radiomics, texture analysis

## Abstract

Malignant middle cerebral artery infarction (mMCAi) is a serious complication of cerebral infarction usually associated with poor patient prognosis. In this retrospective study, we analyzed clinical information as well as non-contrast computed tomography (NCCT) and computed tomography angiography (CTA) data from patients with cerebral infarction in the middle cerebral artery (MCA) territory acquired within 24 h from symptoms onset. Then, we aimed to develop a model based on the radiomics signature to predict the development of mMCAi in cerebral infarction patients. Patients were divided randomly into training (*n* = 87) and validation (*n* = 39) sets. A total of 396 texture features were extracted from each NCCT image from the 126 patients. The least absolute shrinkage and selection operator regression analysis was used to reduce the feature dimension and construct an accurate radiomics signature based on the remaining texture features. Subsequently, we developed a model based on the radiomics signature and Alberta Stroke Program Early CT Score (ASPECTS) based on NCCT to predict mMCAi. Our prediction model showed a good predictive performance with an AUC of 0.917 [95% confidence interval (CI), 0.863–0.972] and 0.913 [95% CI, 0.795–1] in the training and validation sets, respectively. Additionally, the decision curve analysis (DCA) validated the clinical efficacy of the combined risk factors of radiomics signature and ASPECTS based on NCCT in the prediction of mMCAi development in patients with acute stroke across a wide range of threshold probabilities. Our research indicates that radiomics signature can be an instrumental tool to predict the risk of mMCAi.

## Introduction

Malignant middle cerebral artery infarction (mMCAi) is a life-threatening complication that is usually observed in relatively young patients with a large middle cerebral artery (MCA) infarction (Hacke et al., 1996). Its prevalence rate hovers around 10–15% of the total MCA strokes (Ryoo et al., 2004), and around 18–31% of strokes caused by MCA occlusion (Foerch et al., 2004). In mMCAi patients, the early decompressive hemicraniectomy was found effective in reducing the mortality rate and improving the clinical outcome (Vahedi et al., 2007; Jüttler et al., 2014; Back et al., 2015). However, mortality rates of mMCAi can reach up to 80% without surgical intervention (Thomalla et al., 2010; Sakuta et al., 2015).

To the best of our knowledge, there are no standardized parameters that can define mMCAi especially in the early stages. Several clinical and standard visual radiological parameters have been studied as possible predictors for the development of mMCAi (Thomalla et al., 2010). In particular, severe neurological deterioration reflected by high scores on the National Institutes of Health Stroke Scale (NIHSS) was a predictor of fatal cerebral edema (Oppenheim et al., 2000). The occlusion of the internal carotid artery (ICA) in the intracranial segment on computed tomography angiography (CTA) could predict mMCAi (Kucinski et al., 1998). Further, the presence of extensive early ischemic signs (including the involvement of more than 50% of the MCA territory) (Kasner et al., 2001) or hyperdense vessel sign (HVS) on CT imaging (Haring et al., 1999) can also predict mMCAi. However, the positive predictive value of the above-mentioned parameters was modest (Hofmeijer et al., 2008). Thomalla et al. (2010) defined mMCAi as follows: (1) clinical signs of large MCA territory infarction with a NIHSS score >18 and a level of consciousness of ≥1 on item 1a of the NIHSS either on admission or after secondary deterioration; (2) large space-occupying MCA infarction on follow-up MRI or CT occupying at least two-thirds of the MCA territory with compression of ventricles or midline shift; and (3) no other obvious causes for neurological deterioration. While, Shimoyama et al. (2014) defined mMCAi as clinical deterioration, midline shift ≥5 mm, or brain herniation within 48 h of admission.

Accurate prediction of mMCAi can positively impact clinical decisions and patient prognosis. Interestingly, the ratio of intracranial cerebrospinal fluid (CSF) volume to the intracranial volume (ICV) was reported to be independently associated with mMCAi (Kauw et al., 2019). In the same context, an optimal diffusion-weighted magnetic resonance imaging (DWI) cutoff >80 ml could also predict the fulminant course within ≈6 h of the stroke onset (Mlynash et al., 2011). Specifically, a DWI volume >145 ml was predictive of mMCAi when magnetic resonance (MR) imaging was performed at 14 h after stroke onset (Oppenheim et al., 2000). However, MR imaging is not always feasible after stroke onset due to the long examination time and influence of motion artifact. Therefore, uncovering additional mMCAi predictors will be instrumental for achieving accurate diagnosis. Texture analysis of CT images is a noninvasive method that quantifies the heterogeneity of macroscopic tissues which can be indirectly correlated to the heterogeneity of the microscopic tissues (Soni et al., 2019). Recent evidence suggests that texture analysis of CT scans can be valuable for the evaluation of ischemic stroke events (Oliveira et al., 2009). Nevertheless, little is known about the relationship between the radiomics signature based on texture features and the development of mMCAi. In this study, we hypothesize that radiomics signature can serve as an early predictor of mMCAi. To test this hypothesis, we conducted a retrospective study to analyze the value of single radiomics signature as well as the combination of radiomics signature, clinical and standard visual radiological factors in predicting mMCAi. In our study, mMCAi diagnosis was attained upon observing a midline shift >5 mm on follow-up non-contrast computed tomography (NCCT) performed after clinical deterioration or 3 days after stroke (Kauw et al., 2019).

## Materials and Methods

### Patients

**FIGURE 1:**
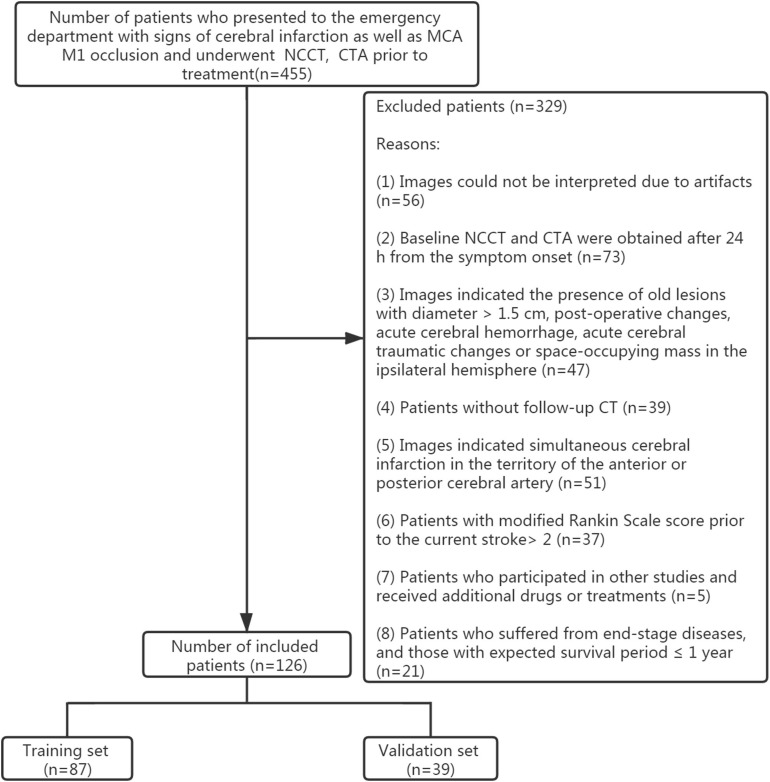
Flowchart of the collection of patients.

This study was approved by the ethics committee of the Zhejiang Provincial People’s Hospital, Affiliated People’s Hospital of Hangzhou Medical College. Owing to the retrospective nature of this study, the need for patients’ informed consent was waived. Patients’ data and radiological findings were obtained from routine clinical and radiological records. The clinical and radiological databases were retrospectively reviewed between January 1, 2017 and November 30, 2019. Inclusion criteria included: (1) patients with symptoms related to cerebral infarction; (2) patients who underwent baseline NCCT and CTA prior to treatment as well as within 24 h from symptoms onset and follow-up NCCT; (3) patients with MCA M1 occlusion proved by baseline CTA and acute MCA infarction proved by follow-up NCCT. Exclusion criteria included: (1) the presence of old lesions with diameter >1.5 cm, post-operative changes, acute cerebral hemorrhage, acute cerebral traumatic changes or space-occupying mass in the ipsilateral hemisphere as indicated by the baseline or follow-up CT; (2) a modified Rankin Scale (mRS) score >2 prior to the current stroke; (3) patients who participated in other studies and those receiving experimental drugs or treatments; (4) patients suffering from end-stage diseases and those with expected survival period ≤1 year; (5) NCCT image artifacts or other reasons that hinder image interpretation; (6) follow-up CT indicating simultaneous cerebral infarction in the territory of the anterior or posterior cerebral artery. Records from 455 patients were initially analyzed. However, only 126 patients were finally included in this study after applying the inclusion and exclusion criteria (Figure 1). Among them, 52 cases were diagnosed with mMCAi. All patients were divided randomly into training (*n* = 87) and validation (*n* = 39) sets according to a 7:3 ratio (Shu et al., 2019).

### Imaging Analysis

NCCT and CTA scans were performed with 640-slice CT (Toshiba, Aquilion ONE TSX-301A). NCCT images were obtained with 1 mm slice thickness, 1mm section gap and 512 × 512 matrix. While CTA images were obtained with 0.5 mm slice thickness, 0.5 mm section gap and 512 × 512 matrix. A total of 50 ml contrast agent (Omnipaque, GE Healthcare) was injected through the elbow vein at 5 ml/s. CTA images of the transverse, coronal and sagittal sections were reconstructed with 3 mm thickness and 2 mm section gap. Maximum intensity projection of CTA images were obtained with 24 mm thickness and 4 mm section gap.

All baseline NCCT, CTA, and follow-up NCCT images were assessed by two experienced neuroradiologists. A total of 30 patients were randomly selected and we analyzed the inter-rater reliability between raters A and B by κ test. In particular, the inter-rater reliability was computed by comparing the results of image analysis from rater B (15 years of experience in interpreting neurological CT scans) and the image analysis from rater A (10 years of experience in interpreting neurological CT scans). A κ test value >0.75 was considered to be a good agreement.

The status of leptomeningeal collaterals was assessed with CTA images. Collaterals were graded on a scale of 0–3 as previously described (Tan et al., 2009; Brunner et al., 2014): (0) absence of collaterals filling to the occluded MCA territory; (1) collaterals filling ≤50 and >0% of the occluded MCA territory; (2) collaterals filling >50 and <100% of the occluded MCA territory; (3) 100% collaterals filling of the occluded MCA territory.

Based on the baseline NCCT, CTA images, as well as scoring criteria, we were able to compute the standard visual analysis of ASPECTS on NCCT, HVS of MCA, collaterals and occlusion of ICA in the intracranial segment on CTA images. Based on the baseline NCCT as well as follow-up NCCT images, we were able to compute the standard visual analysis of information related to hemorrhage transformation.

### Association Between Texture Analysis and mMCAi

Using the baseline NCCT images, we performed the regions of interest (ROI) segmentation of the entire MCA territory manually on a 3D-volume of interest using the ITK-SNAP software^1^ (Supplementary Figures S1A,B,C). The inter-rater reliability on ROI segmentation of MCA territory was computed by comparing the measurement from rater B (15 years of experience in interpreting neurological CT scans) and the measurement from rater A (10 years of experience in interpreting neurological CT scans) in 30 randomly selected patients by κ test. Images underwent preprocessing with AK software (Artificial Intelligence Kit V3.0.0.R, GE Healthcare), which included image interpolation, intensity normalization, and gray-level discretization as described previously (Shu et al., 2019). Next, we calculated the texture features including histogram, formfactor, haralick, run-length matrix (RLM), gray-level co-occurrence matrix (GLCM), and gray-level size zone matrix (GLSZM) with AK software (Artificial Intelligence Kit V3.0.0.R, GE Healthcare). Prior to feature selection, a total of 396 imaging features were extracted for each patient and the extracted texture features were then standardized (Shu et al., 2019). Dimension reduction was performed using analysis of variance and Mann–Whitney *U* test and then we performed a correlation test to reduce data redundancy. Finally, least absolute shrinkage and selection operator (LASSO) was used to further select significant features.

Multivariate logistic regression was undertaken to construct a model of radiomics signature based on the remaining features. Thereafter, we calculated the radiomics score (rad-score) for every patient in both the training and validation sets using the formula constructed in the training set. The predictive efficiency of radiomics signature in both the training and validation sets was then evaluated using the area under the receiver-operator characteristic (ROC) curve (AUC). Calibration of radiomics signature in the prediction of mMCAi was assessed with a calibration curve. Further, decision curve analysis (DCA) was used to assess the clinical efficiency of radiomics signature in predicting mMCAi through calculating the net benefit across a spectrum of threshold probabilities.

### Assessment of the Prediction Model for mMCAi Based on the Combination of Radiomics Signature, Clinical and Standard Visual Radiological Factors

In the training set, different analyses were carried out on each potential predictor (gender, age, baseline NIHSS, hypertension, diabetes mellitus, hyperlipidemia, atrial fibrillation, smoking, alcohol abuse, ASPECTS on NCCT, HVS of MCA, hemorrhage transformation, collaterals, side of the MCA M1 occlusion, and occlusion of ICA in the intracranial segment) to select predictors associated with mMCAi. Consequently, multivariable logistic regression analysis was applied to develop a prediction model for mMCAi. The calibration of the developed model was assessed with a calibration curve and a ROC curve was used to evaluate the discriminability of the new model. Finally, the efficiency of our model in predicting mMCAi was assessed by DCA.

### Statistical Analysis

Statistical analyses were performed using IBM SPSS Statistics (version 21.0) and Microsoft R Open (version 3.3.1). All metric and normally distributed variables were reported as mean ± standard deviation; non-normally distributed variables as median (interquartile range). Categorical variables were presented as numbers (percentages). The κ test was used to assess the inter-rater reliability for image analysis on NCCT, CTA images and ROI segmentation of MCA territory. *T*-test, Chi-square, Fisher’s exact, and Mann–Whitney *U* test were used to identify variables associated with mMCAi. Then, LASSO logistic regression model with penalty parameter tuning was conducted by 10-fold cross-validation based on the minimum criteria to select the most valuable predictive features. Backward stepwise selection was applied through a likelihood ratio test and Akaike’s information criterion (AIC) as the stopping rule. Multivariate logistic regression analysis was used to construct a prediction model. Calibration plots were performed on the “rms” package (Microsoft R Open; version 3.3.1). ROC curve was used to evaluate the discriminability of the prediction model. DCA was performed using the “dca.R.” (Microsoft R Open; version 3.3.1). A *P-*value < 0.05 was considered to be statistically significant.

## Results

### Patients’ Characteristics

The inter-rater agreement between our two neuroradiologists ranged from 0.783 to 0.843 which indicated a favorable inter-rater reproducibility. With exception to NIHSS, there were no statistical differences between the training and validation sets in the clinical and standard visual radiological variables (Table 1). In the training set, we observed statistical differences in the baseline NIHSS, ASPECTS on NCCT, ICA occlusion, and rad-score between the mMCAi and non-mMCAi groups (Table 2). Similar results were also observed in the validation set. In addition, a statistical difference in HVS of MCA was also observed between mMCAi and non-mMCAi groups in the validation set (Table 2).

**TABLE 1:** Demographic characters and clinical features of patients in the training and validation sets.

Variable	Training (*n* = 87)	Validation (*n* = 39)	*P*-value
Male sex, *n* (%)	54(62.07%)	25(64.10%)	0.828
Age, *y*; mean ± SD	72.45 ± 13.96	71.31 ± 10.80	0.180
Baseline NIHSS, median (IQR)	20(15–25)	17(13–21)	0.029
Hypertension, *n* (%)	62(71.26%)	21(53.85%)	0.069
Diabetes mellitus, *n* (%)	19(21.84%)	4(10.26%)	0.141
Hyperlipidemia, *n* (%)	20(22.99%)	7(17.95%)	0.641
Atrial fibrillation, *n* (%)	41(47.13%)	21(53.85%)	0.564
Smoking, *n* (%)	31(35.63%)	11(28.21%)	0.540
Alcohol abuse, *n* (%)	19(21.84%)	7(17.95%)	0.812
ASPECTS on NCCT, median (IQR)	7(5–9)	7(4–9)	0.932
HVS of MCA, *n* (%)	49(56.32%)	19(48.72%)	0.446
Hemorrhage transformation, *n* (%)	33(37.93%)	18(46.15%)	0.435
Collateral score, median (IQR)	1(1–1)	1(1–1)	0.746
Right side of the MCA M1 occlusion, *n* (%)	42(48.28%)	23(58.97%)	0.336
ICA occlusion, *n* (%)	36(41.38%)	15(38.46%)	0.845

*SD, standard deviation; NIHSS, National Institutes of Health Stroke Scale; IQR, interquartile range; ASPECTS, Alberta Stroke Program Early CT Score; NCCT, non-contrast computed tomography; HVS, hyperdense vessel sign; MCA, middle cerebral artery; ICA, internal carotid artery.*

**TABLE 2:** Comparison between mMCAi and non-mMCAi patients in the training and validation sets.

Variable	Training (*n* = 87)			Validation (*n* = 39)		
		
	mMCAi (*n* = 36)	Non-mMCAi (*n* = 51)	*P-*value	mMCAi (*n* = 16)	Non-mMCAi (*n* = 23)	*P-*value
Male sex, *n* (%)	22(61.11%)	32(62.75%)	0.878	10(62.50%)	15(65.22%)	0.864
Age, *y*; mean ± SD	75.14 ± 12.60	70.55 ± 14.67	0.403	72.06 ± 10.42	70.78 ± 11.26	0.799
Baseline NIHSS, median (IQR)	23(19.25–27)	17(14–21)	0.001	18.50(16.25–21.75)	16(7–18)	0.028
Hypertension, *n* (%)	26(72.22%)	36(70.59%)	0.869	7(43.75%)	14(60.87%)	0.342
Diabetes mellitus, *n* (%)	9(25.00%)	10(19.61%)	0.604	1(6.25%)	3(13.04%)	0.631
Hyperlipidemia, *n* (%)	11(30.56%)	9(17.65%)	0.199	3(18.75%)	4(17.39%)	0.915
Atrial fibrillation, *n* (%)	21(58.33%)	20(39.22%)	0.087	10(62.50%)	11(47.83%)	0.516
Smoking, *n* (%)	9(25.00%)	22(43.14%)	0.112	7(43.75%)	4(17.39%)	0.146
Alcohol abuse, *n* (%)	6(16.67%)	13(25.49%)	0.432	5(31.25%)	2(8.70%)	0.101
ASPECTS on NCCT, median (IQR)	5(1.25–8)	8(5–10)	<0.001	3.50(1.25–5.75)	8(7–10)	< 0.001
HVS of MCA, *n* (%)	23(63.89%)	26(50.98%)	0.276	12(75.00%)	7(30.43%)	0.010
Hemorrhage transformation, *n* (%)	13(36.11%)	20(39.22%)	0.825	8(50.00%)	10(43.48%)	0.752
Collateral score, median (IQR)	1(1–1)	1(1–1)	0.051	1(1–1)	1(1–1)	0.767
Right side of the MCA M1 occlusion, *n* (%)	18(50.00%)	24(47.06%)	0.830	12(75.00%)	11(47.83%)	0.111
ICA occlusion, *n* (%)	20(55.56%)	16(31.37%)	0.029	10(62.50%)	5(21.74%)	0.018
Rad score, mean ± SD	1.82 ± 4.12	−1.65 ± 1.64	0.020	1.59 ± 3.67	−1.02 ± 1.03	0.001

*SD, standard deviation; NIHSS, National Institutes of Health Stroke Scale; IQR, interquartile range; ASPECTS, Alberta Stroke Program Early CT Score; NCCT, non-contrast computed tomography; HVS, hyperdense vessel sign; MCA, middle cerebral artery; ICA, internal carotid artery.*

### Assessment of Radiomics Signature

**FIGURE 2:**
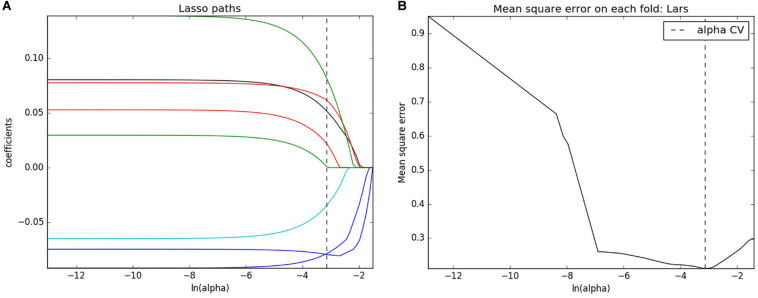
Texture feature selection. **(A)** LASSO coefficient profiles of texture features. **(B)** Mean square error on each fold for texture feature selection with LASSO.

Analysis of variance and Mann–Whitney *U* test selected 201 features that can be associated with the development of mMCAi. Following spearman correlation analysis, those features were further narrowed down to 17. Next, following LASSO regression, the number of texture features was further reduced to 8 (Figures 2A,B and Supplementary Figures S2A,B). Finally, multivariate logistic regression was used to build the prediction model in which only three texture features remained (ClusterShade_AllDirection_offset4_SD, Compactness2 and LongRunLowGreyLevelEmphasis_angle45_ offset4; Supplementary Table S1). Subsequently, rad-score was obtained from the LASSO model to reflect the risk of mMCAi (Supplementary Material). Interestingly, the radiomics signature showed a favorable predictive efficacy, with an AUC of 0.866 [95% confidence interval (CI), 0.792–0.940] and 0.802 [95% CI, 0.647–0.956] in the training and validation sets, respectively (Supplementary Figures S3A,B). DCA validated the clinical power of the radiomics signature in mMCAi prediction across a wide range of threshold probabilities within 0.05 to 1 in the training set and 0.24 to 1 in the validation set (Supplementary Figures S4A,B). Likewise, the calibration curves exhibited favorable calibration power in the training and validation sets (Supplementary Figures S5A,B).

### Efficacy of the Prediction Model in Predicting mMCAi

**FIGURE 3:**
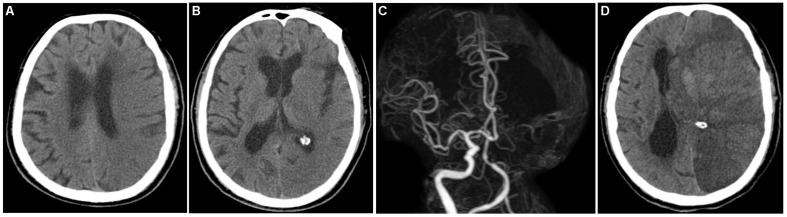
A 70-year-old male patient who underwent baseline NCCT and CTA scan at 3.7 h after onset of left hemispheric stroke and follow-up CT 18.2 h from symptoms onset. **(A,B)** Baseline NCCT demonstrating a left hemispheric stroke with ASPECTS of 2. **(C)** CTA showing left MCA M1 and ICA occlusion. **(D)** Follow-up CT demonstrating the evolution of mMCAi with midline shift of 6.2 mm and hemorrhage transformation.

**FIGURE 4:**
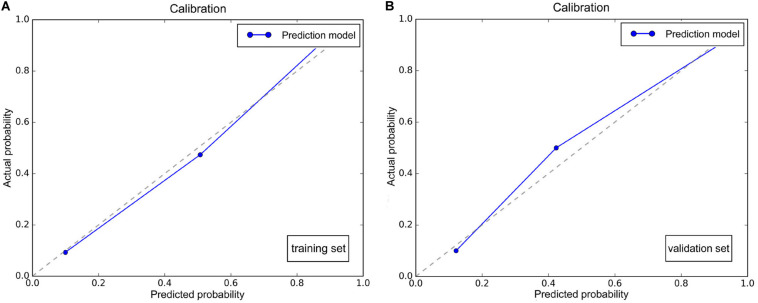
**(A)** Calibration plot of the prediction model based on ASPECTS on NCCT and rad-score in the training set. **(B)** Calibration plot of the prediction model based on ASPECTS on NCCT and rad-score in the validation set.

**FIGURE 5:**
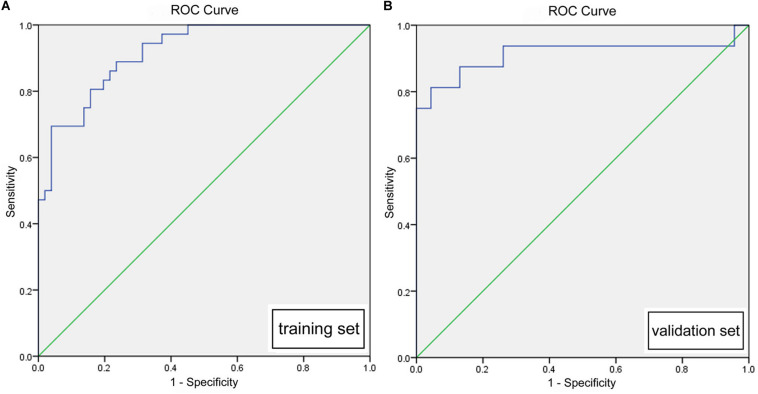
**(A)** ROC of the prediction model based on ASPECTS on NCCT and rad-score in the training set (0.917, [0.863, 0.972]). **(B)** ROC of the prediction model based on ASPECTS on NCCT and rad-score in the validation set (0.913, [0.795, 1]).

**FIGURE 6:**
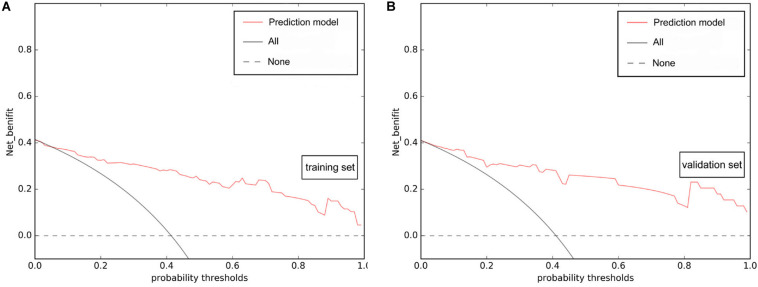
DCA of the prediction model based on ASPECTS on NCCT and rad-score. The prediction model presented better net benefit gains compared to the “reduction all” or “reduction none” strategies across a wide range of threshold probabilities within 0.067–1 in the training set **(A)** and 0.046–1 in the validation set **(B)**.

Multivariate logistic regression analysis identified rad-score, calculated from the radiomics signature, as well as ASPECTS on NCCT as independent factors that can predict mMCAi (Figures 3A–D). A non-significant Hosmer-Lemeshow test (*P* = 0.919) showed good calibration in the training set. In both the training and validation sets, calibration curves of the developed model exhibited good calibration power (Figures 4A,B). The detection accuracy for our model was 0.917 [95% CI, 0.863–0.972] and 0.913 [95% CI, 0.795–1] in the training and validation sets, respectively (Figures 5A,B). Further, DCA validated the clinical usefulness of the prediction model in differentiating mMCAi from non-mMCAi patients across a wide range of threshold probabilities within 0.067–1 in the training set and 0.046–1 in the validation set (Figures 6A,B). It is worth mentioning that analyzing the efficacy of ASPECTS on NCCT alone demonstrated a medium diagnostic accuracy for mMCAi prediction, with AUC of 0.726 [95% CI, 0.620–0.816], sensitivity of 0.389, and specificity of 0.961 and the optimal cut-off value of ASPECTS on NCCT was 3 with Youden index (0.350).

## Discussion

In this study, we developed a new model that can predict the development of mMCAi in MCA stroke patients. The development of mMCAi is a devastating event with mortality rates reaching up to 80% in untreated cases (Thomalla et al., 2010; Sakuta et al., 2015). The pathophysiology of mMCAi is complex, but it can be traced back to hemodynamic and metabolic changes that lead to destruction of the blood-brain barrier (Gasche and Copin, 2003; Heo et al., 2005; Walcott et al., 2011). Early detection of mMCAi and timely management (within 48 h) with a decompressive surgery may improve the patient prognosis (Cho et al., 2003; Thomalla et al., 2010). Therefore, developing a strategy that enables early prediction of mMCAi is urgently required to enable timely intervention.

In this study, we evaluated the value of texture features in predicting mMCAi. An image texture is a representation of intensity, distribution, and inter-relationships between pixels, which may not be noticed by naked eye. Texture analysis can measure the disease heterogeneity (through parameters like entropy, kurtosis, and pixel distribution that can be correlated with angiogenesis, cellular density, and necrosis) in a noninvasive manner which can ultimately lead to a better understanding of disease biology (Miles et al., 2013; Raja et al., 2016).

To date, research regarding the beneficial role of texture analysis in cerebral infarction is still scarce (Sikiö et al., 2015). Nevertheless, texture analysis was found beneficial in identifying the presence of previous stroke lesions on MR images (Ortiz-Ramón et al., 2019). Further, it was also instrumental in the early identification of cerebral infarction and quantification of involved areas (Oliveira et al., 2009). Compared to visual inspection, texture analysis was superior in identifying acute ischemic stroke patients with higher risk of hemorrhage transformation (Kassner et al., 2009). However, the relationship between radiomics signature based on texture features and the development of mMCAi remains obscure. In this study, we aimed to analyze the efficacy of radiomics in predicting the development of mMCAi. Our results demonstrated that the radiomics signature based on texture features can indeed be used as a valuable tool for identifying mMCAi. The radiomics signature showed a favorable predictive efficacy, with an AUC of 0.866 and 0.802 in the training and validation sets, respectively.

In the recent years, the use of multi-parameter analysis, which combines individual markers to form comprehensive marker panels, has become a prospective tool for a better diagnostic performance (Birkhahn et al., 2007). Thus, in our study, we combined the clinical, standard visual radiological factors and radiomics signature to develop a new model that can predict mMCAi. Indeed, we found that rad-score based on the radiomics signature and ASPECTS on NCCT were independent predictors of mMCAi development. The optimal cut-off value of ASPECTS was 3 when using single ASPECTS to predict mMCAi, with a sensitivity and specificity of 0.389 and 0.961, respectively. A lower ASPECTS value was correlated with the development of mMCAi. In agreement, MacCallum et al. (2014) previously confirmed the value of ASPECTS in detecting mMCAi. However, they demonstrated that the optimal cut-off value of ASPECTS was 7, with 50% sensitivity and 86% specificity. The detection accuracy for our model, based on radiomics signature and ASPECTS on NCCT, was 0.917 and 0.913 in the training and validation sets, respectively. Therefore, the addition to radiomics signature increased the strength of our prediction model. Ong et al. (2017) developed a risk prediction score using the clinical and radiological parameters observed within 24 h of ictus to predict mMCAi. However, his model showed a moderate predictive performance with an AUC of 0.76 [95% CI, 0.68–0.82] in the derivation set, and 0.75 [95% CI, 0.72–0.77] in the bootstrapping validation set. Further, Cheng et al. (2020) constructed a mMCAi prediction model utilizing a modified enhanced detection of edema in malignant anterior circulation stroke (EDEMA) score based on clinical and radiological variables. The discriminative ability of that model was 0.80 [95% CI, 0.76–0.84]. Additionally, Shimoyama et al. (2014) used the DASH score to assess the risk of mMCAi development in large MCA infarctions, with an AUC of 0.88 [95% CI, 0.82–0.94]. Interestingly, adding the CT-based texture feature analysis increased the strength of our proposed model compared to the above-mentioned methods. To the best of our knowledge, this is the first study to evaluate and internally validate a model composed of rad-score and ASPECTS on NCCT to detect mMCAi. This model showed good calibration and differentiation capabilities in both the training and validation sets.

It should be noted that there are few limitations in our study. First, the retrospective nature of our study cannot negate the risk of information and selection bias. However, results obtained from this study enabled the development of a preliminary detection model. Second, the sample size of the validation model was relatively small. Therefore, future multi-center prospective studies with larger sample size should be to validate the accuracy of our model. Third, we did not use the clinical parameters to define mMCAi. Although some previous studies combined the clinical parameters with imaging information for defining mMCAi, we believe that the quantitative measurement of midline shift was sufficient to identify mMCAi (Walcott et al., 2014; Kauw et al., 2019). Finally, owing to the difficulty of recognizing the real extent of ischemic stroke by naked eye, especially in the early stage, we designated the entire MCA territory as a ROI. In spite of those limitations, a prediction model has been constructed by combining radiomics signature and ASPECTS on NCCT. This model can facilitate the early and accurate prediction of mMCAi evolution and can be helpful in the development of clinical decisions. We expect that our model will be instrumental for the accurate prediction of mMCAi. Future prospective multi-center studies will aim to validate the efficiency of this model.

## Data Availability Statement

The raw data supporting the conclusions of this article will be made available by the authors, without undue reservation.

## Ethics Statement

The studies involving human participants were reviewed and approved by the ethics committee of the Zhejiang Provincial People’s Hospital, Affiliated People’s Hospital of Hangzhou Medical College. Written informed consent for participation was not required for this study in accordance with the national legislation and the institutional requirements.

## Author Contributions

XW and XG designed the study. XHe, ZS, XHu, JC, and HJ performed the data acquisition and analysis. XW, XG, YX, and YL drafted and wrote the manuscript. All authors contributed to the article and approved the submitted version.

## Conflict of Interest

The authors declare that the research was conducted in the absence of any commercial or financial relationships that could be construed as a potential conflict of interest.

## Footnotes

1 www.itksnap.org

## Supplementary Material

FIGURE S1 ROI segmentation of the MCA territory in a 65-year-old male patient. **(A)** Baseline NCCT. **(B)** ROI segmentation of MCA territory on the transverse section image using ITK-SNAP software. **(C)** 3D reconstruction of ROI.

FIGURE S2 (A,B) figures show the correlation heatmap between features which were selected using LASSO in the training and testing sets, respectively.

FIGURE S3 **(A)** ROC of the radiomics signature based on texture features in mMCAi prediction in the training set (AUC [95% confidence interval], 0.866 [0.792, 0.940]). **(B)** ROC of the radiomics signature based on texture features in mMCAi prediction in the validation set (0.802, [0.647, 0.956]).

FIGURE S4 DCA indicating that radiomics signature is valuable in the prediction of mMCAi when the threshold probability was within a range from 0.05 to 1 in the training set **(A)** and 0.24 to 1 in the validation set **(B)**.

FIGURE S5 **(A)** Calibration plot of radiomics signature based on texture features in the prediction of mMCAi in the training set. **(B)** Calibration plot of radiomics signature based on texture features in the prediction of mMCAi in the validation set.

TABLE S1 The classification and calculation formula of texture features in three subgroups.
