# Supplementary figures and images for: Prediction of Malignant Acute Middle Cerebral Artery Infarction via Computed Tomography Radiomics
Source: Front Neurosci. 2020 Jul 7;14:708. doi: 10.3389/fnins.2020.00708 (PMC7358521; doi:10.3389/fnins.2020.00708)

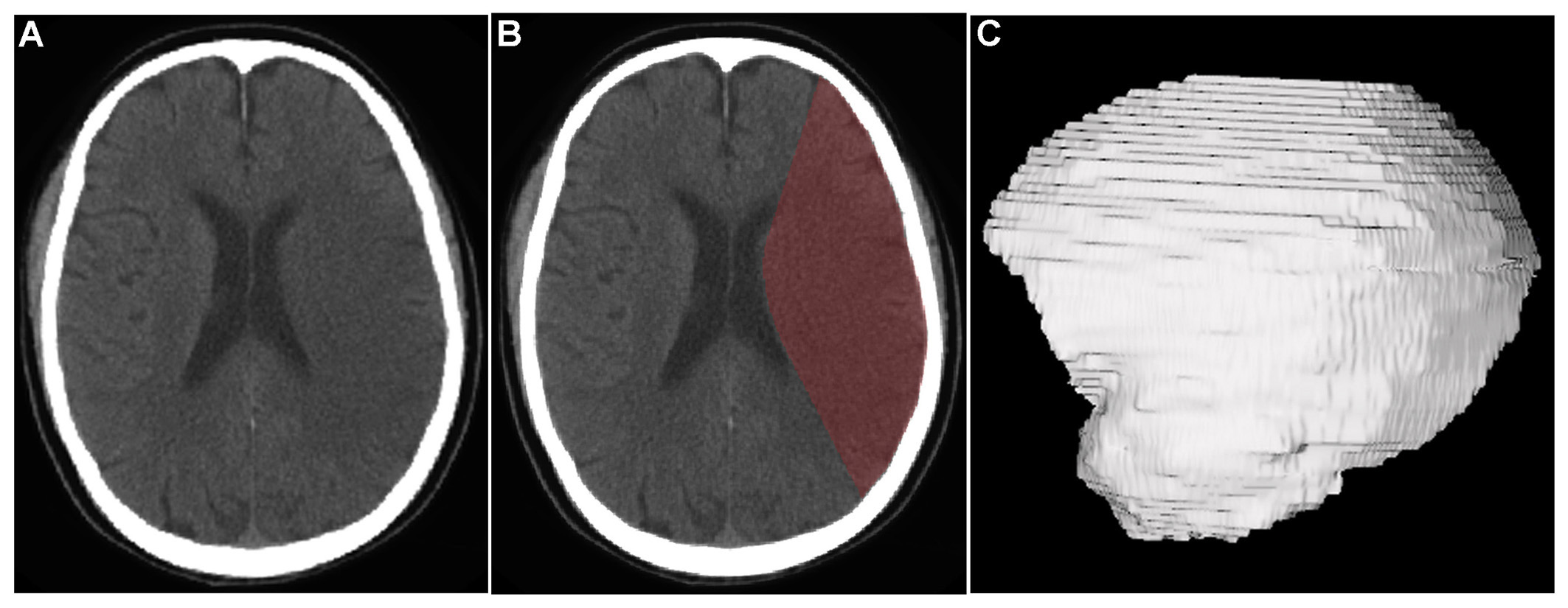

Supplement: FIGURE S1 — ROI segmentation of the MCA territory in a 65-year-old male patient. (A) Baseline NCCT. (B) ROI segmentation of MCA territory on the transverse section image using ITK-SNAP software. (C) 3D reconstruction of ROI. [file Image_1.jpg]

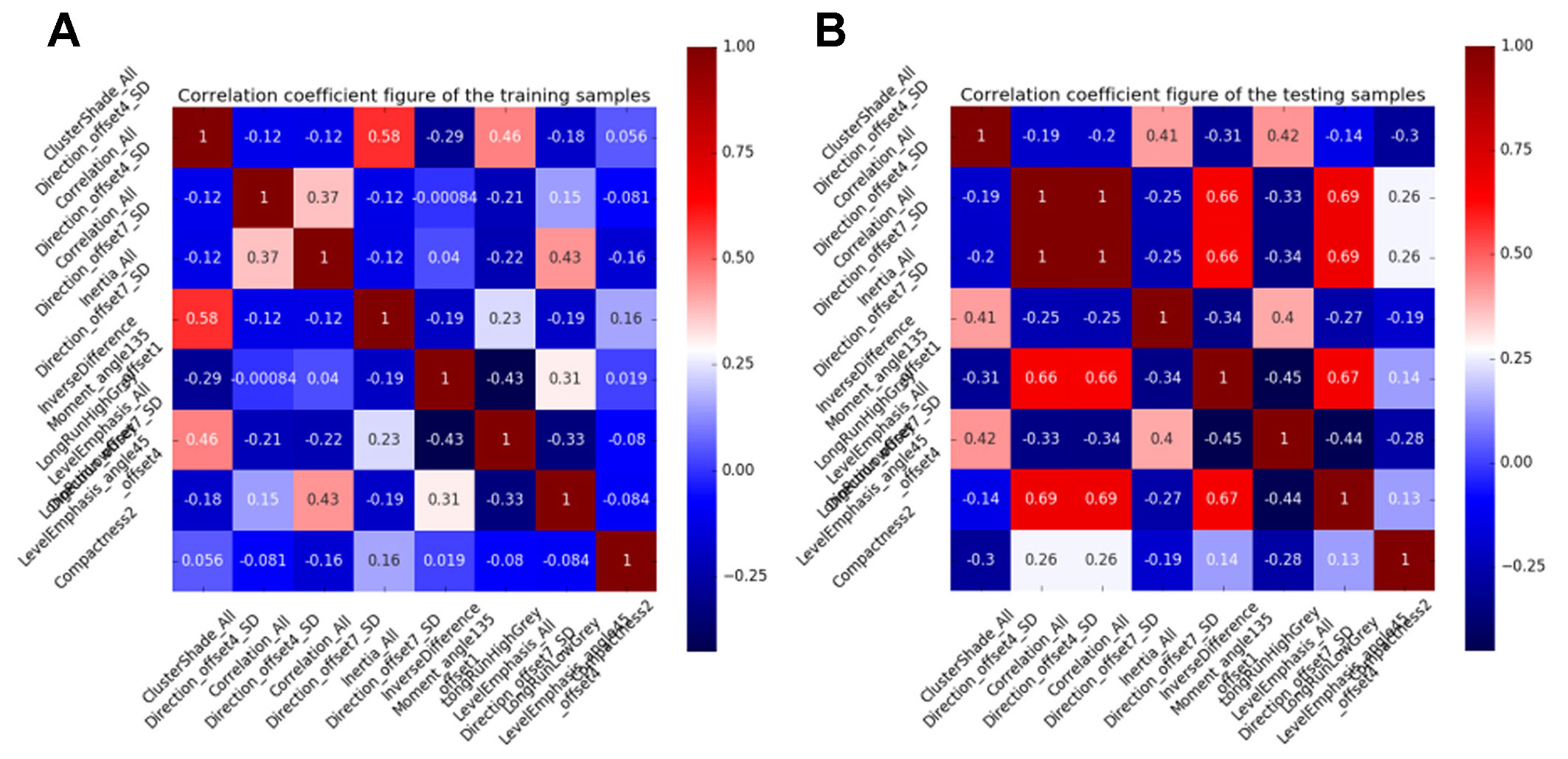

Supplement: FIGURE S2 (A,B) — figures show the correlation heatmap between features which were selected using LASSO in the training and testing sets, respectively. [file Image_2.jpg]

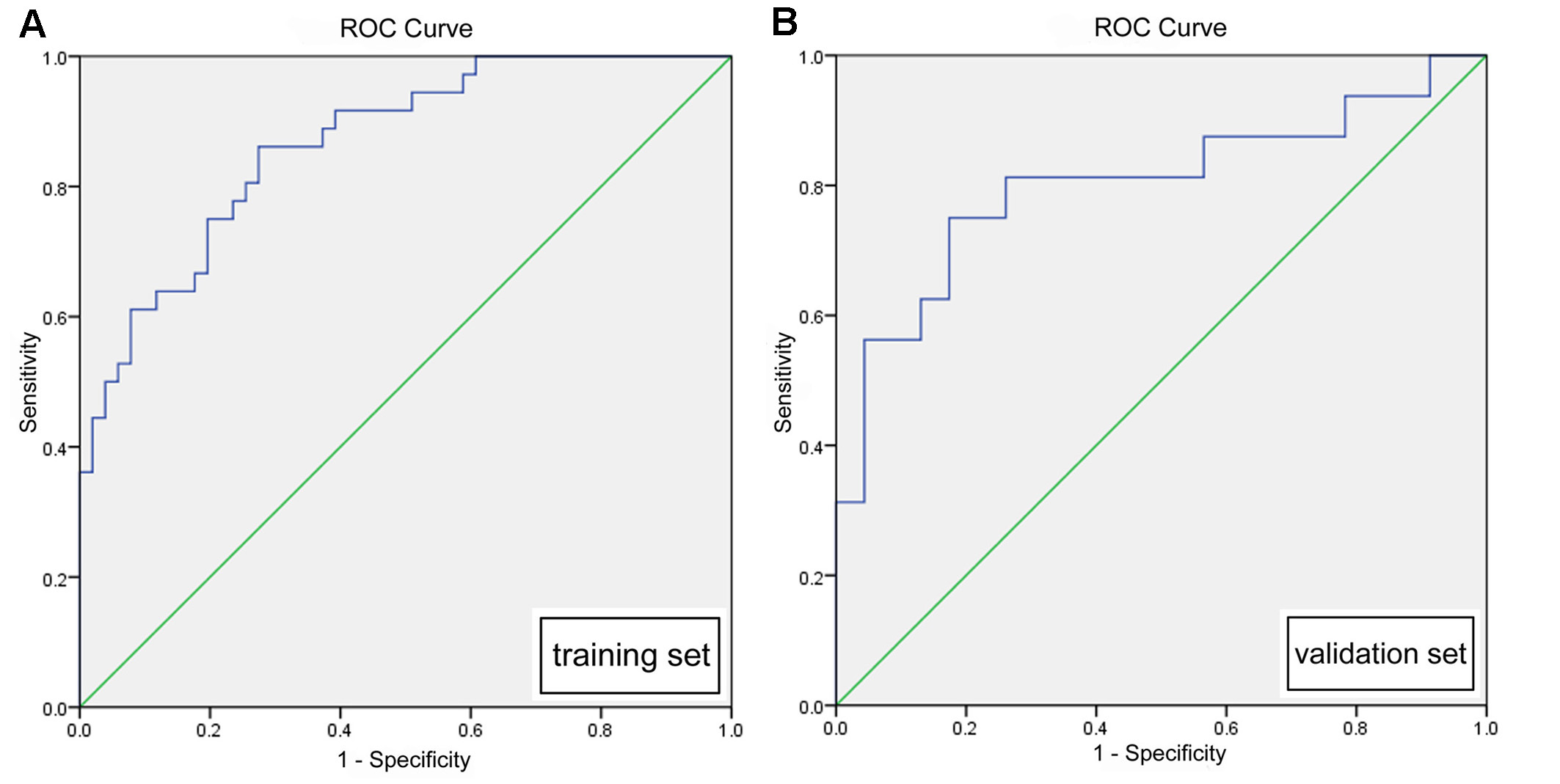

Supplement: FIGURE S3 — (A) ROC of the radiomics signature based on texture features in mMCAi prediction in the training set (AUC [95% confidence interval], 0.866 [0.792, 0.940]). (B) ROC of the radiomics signature based on texture features in mMCAi prediction in the validation set (0.802, [0.647, 0.956]). [file Image_3.jpg]

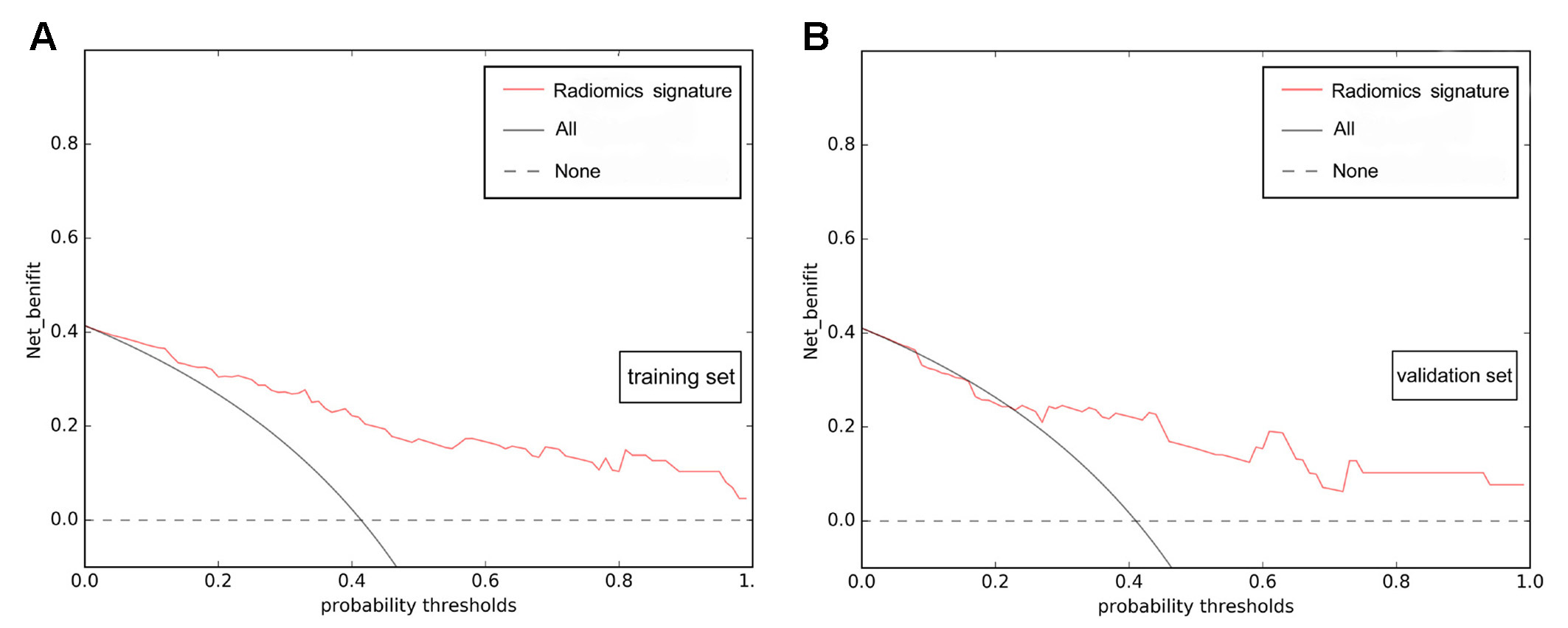

Supplement: FIGURE S4 — DCA indicating that radiomics signature is valuable in the prediction of mMCAi when the threshold probability was within a range from 0.05 to 1 in the training set (A) and 0.24 to 1 in the validation set (B). [file Image_4.jpg]

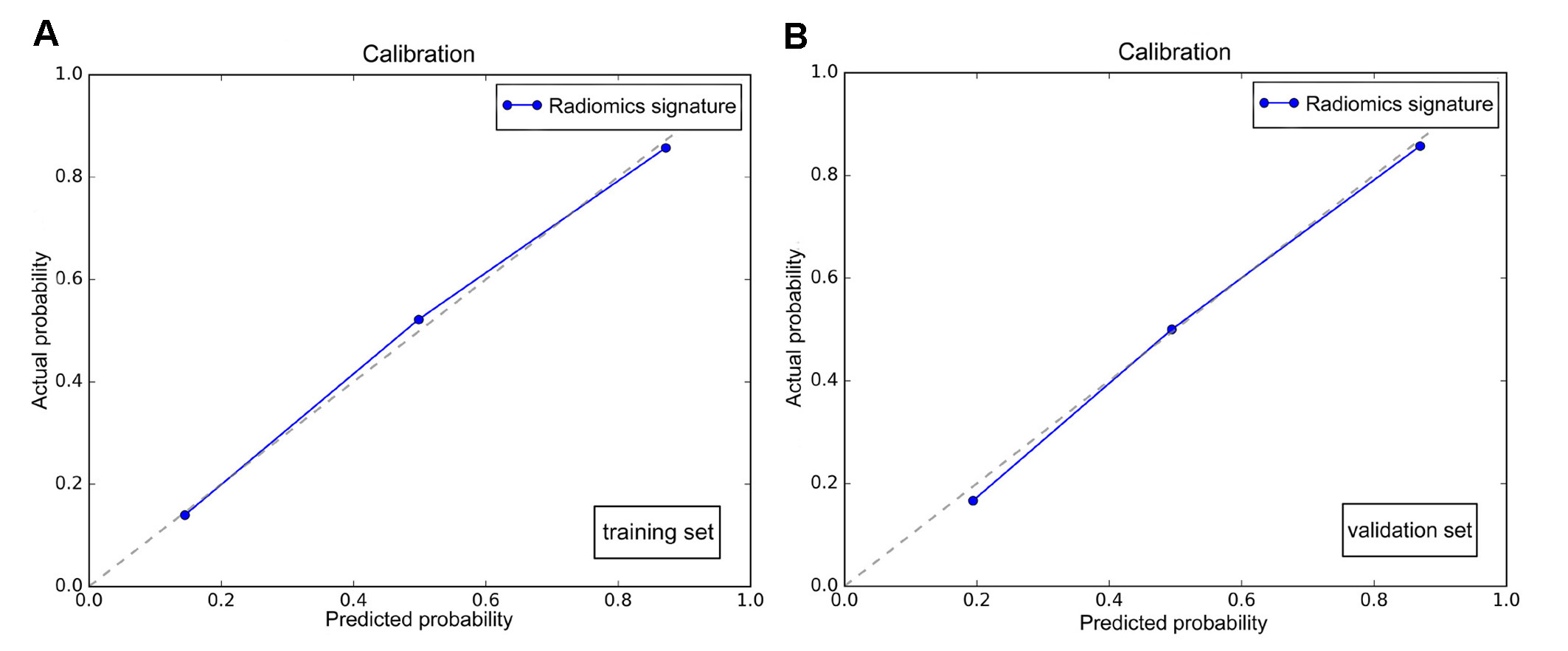

Supplement: FIGURE S5 — (A) Calibration plot of radiomics signature based on texture features in the prediction of mMCAi in the training set. (B) Calibration plot of radiomics signature based on texture features in the prediction of mMCAi in the validation set. [file Image_5.jpg]
