# Supplementary material for: Prediction of Malignant Acute Middle Cerebral Artery Infarction via Computed Tomography Radiomics
Source: Front Neurosci. 2020 Jul 7;14:708. doi: 10.3389/fnins.2020.00708 (PMC7358521; doi:10.3389/fnins.2020.00708)
Supplement: TABLE S1 — The classification and calculation formula of texture features in three subgroups. [file Table_1.DOCX]

**Supplementary Material**

**Details of absolute shrinkage and selection operator (LASSO) algorithm**

LASSO is a powerful algorithm for regression analysis with high dimensional predictors. In our study, we used LASSO to select the most important predictive features in the training set.

The LASSO algorithm shrinks some coefficients and reduces others to exactly 0 via the absolute constraint. Thus, LASSO is an outstanding method for feature selection by retaining the good features of both subset selection and ridge regression. In this study, there were 8 most valuable texture features left following LASSO. The multivariate logistic regression was used to build the model of LASSO in which only 3 texture features remained (ClusterShade_AllDirection_offset4_SD, Compactness2, and LongRunLowGreyLevelEmphasis_angle45_offset4). Details of 3 remaining texture features are shown in **Supplementary Table S1**. At last, a formula was generated using a linear combination of selected features that were weighted by their respective LASSO coefficients. The “glmnet” package in R statistical software version 3.3.1 was used for LASSO logistic regression model analysis. The specific formula is as follows:

**calculation formula:**

Rad-score = -0.21319206-1.37916006×ClusterShade_AllDirection_offset4_SD

+0.86970618×Compactness2

+2.78417129×LongRunLowGreyLevelEmphasis_angle45_offset4

**Supplementary Table S1 |** The classification and calculation formula of texture features in three subgroups.

| **Category** | **Feature** | | **Formula** | **Description** |
| --- | --- | --- | --- | --- |
|  | | | | |
| RLM | | LongRunLowGreyLevelEmphasis_angle45_offset4 | $LRLGE(\theta)=\frac{1}{n_{r}}\sum_{j=i}^{N} \sum_{i=1}^{M} \frac{p(i,j,\theta)j^{2}}{i^{2}}$ | The grey level run-length matrix (RLM) 𝐏𝐫(𝐢, 𝐣 \| 𝛉 ) is defined as the numbers of runs with pixels of gray level *i* and run length *j* for a given direction θ. RLMs is generated for each sample image segment having directions (0°,45°,90° &135°), then the following ten statistical features were derived: Short Run Emphasis, Long Run Emphasis, Grey Level Non-uniformity, Run Length Non-uniformity, Low Grey Level Run Emphasis, High Grey Level Run Emphasis, Short Run Low Grey Level Emphasis, Short Run High Grey Level Emphasis, Long Run Low Grey Level Emphasis and Long Run High Grey Level Emphasis. |
| Histogram parameter | | ClusterShade_AllDirection_offset4_SD | $\sum_{i,j} ((i-\mu)+(j-\mu))^{3}g(i,j)$  Where i, j are the spatial coordinates of g (i, j) | Cluster analysis is the task of grouping a set of objects in such a way that objects in the same group (cluster) are more similar (in some sense or another) to each other than to those in other groups (clusters). It is a common technique for statistical data analysis. |
| Form Factor | | Compactness2 | $C\text{om}pactness2=36\pi\frac{V^{2}}{A^{3}}$  Where *V* is the the volume of interest.  Where *A* is the surface area of the volume of interest. | Form Factor Parameters include descriptors of the three-dimensional size and shape of the lesion region. |
